# Metabolic Syndrome and the Risk of Preclinical Heart Failure: Insights after 17 Years of Follow-Up from the STANISLAS Cohort

**DOI:** 10.1159/000523717

**Published:** 2022-02-23

**Authors:** Abhinav Sharma, Amir Razaghizad, João Pedro Ferreira, Jean-Loup Machu, Erwan Bozec, Nicolas Girerd, Patrick Rossignol, Faiez Zannad

**Affiliations:** ^a^INSERM CIC-P 1433, CHRU de Nancy, INSERM U1116, FCRIN INI-CRCT (Cardiovascular and Renal Clinical Trialists), Université de Lorraine, Nancy, France; ^b^Division of Cardiology, McGill University Health Centre, Montreal, Québec, Canada; ^c^Department of Physiology, University of Porto, Porto, Portugal

**Keywords:** Metabolic syndrome, Heart failure, Preclinical heart failure

## Abstract

**Background:**

We used data from people initially free of clinical cardiovascular disease to evaluate the association between metabolic syndrome (MS) and incident preclinical heart failure (pHF).

**Methods and Results:**

STANISLAS was a familial, single-center, longitudinal prospective cohort study composed of 1,006 families from Nancy, France (median follow-up, 17 years [1993–2016]). Age- and sex-adjusted logistic regression and inverse probability weighting models were used to evaluate the association between MS and pHF, which was defined by diastolic dysfunction, atrial enlargement, ventricular hypertrophy, or elevated natriuretic peptides. Among 944 people who were adults at the first and final visit, those with baseline MS were more likely to be older (63 vs. 61 vs. 59 years of age) and male (73% vs. 55% vs. 45%) compared to people who developed incident MS and people who had no baseline MS, respectively. Furthermore, compared to people without baseline MS, the risk of pHF was numerically larger among people with baseline MS (adjusted odds ratio [aOR] 2.27, 95% CI: 1.07–4.81) and people who developed incident MS (aOR 1.56, 95% CI: 1.00–2.43). Concerning the metabolic determinants of MS, the risk of pHF was most elevated in people with baseline hypertension (aOR 3.19, 95% CI: 1.80–5.63) and elevated waist circumference (aOR 2.59, 95% CI: 1.47–4.57).

**Conclusion:**

Overall, HF is an important public health concern given the high risk of mortality when patients with MS or elevated fasting glucose become established with the disease. Early aggressive lifestyle modification and medical intervention among patients free of cardiovascular disease with an obese-hypertensive phenotype may be warranted to prevent HF development.

## Introduction

There is a high prevalence of metabolic syndrome (MS) among patients with preclinical heart failure (HF) [1]. Although these diseases are often coprevalent, the association between MS and the risk of incident preclinical HF is unclear. Our group previously described an association between elevated waist circumference and an increased risk of diastolic dysfunction development [2]. The findings suggested that targeting MS or its metabolic determinants through lifestyle or medical intervention could be an effective strategy to decrease the incidence of preclinical HF. To address this knowledge gap, we used data from participants initially free of cardiovascular disease in the STANISLAS cohort (Suivi Temporaire Annuel Non-Invasif de la Santé des Lorrains Assurés Sociaux: NCT01391442) to evaluate the epidemiological association between baseline or incident MS [3], and its metabolic determinants, on preclinical HF as defined by the American Heart Association.

## Methods

STANISLAS was designed as a familial, single-center, prospective longitudinal cohort study composed of 1,006 families from Nancy, France, who were recruited between 1993 and 1995. The families were healthy and free of any acute and/or chronic illnesses at recruitment. From 2011 to 2016, STANISLAS participants were revisited for a fourth examination (V4) at our department as previously described [4]. Echocardiographic assessments were conducted at the baseline (V1) and V4 examination, and biological biomarkers were routinely assessed.

Preclinical HF was defined at the V4 examination by the presence of diastolic dysfunction [5, 6], left atrial enlargement, left ventricular hypertrophy, or elevated N-terminal pro-brain natriuretic peptides (NTproBNP) >125 pg/mL. This definition of preclinical HF captures echocardiographic structural features and biomarkers (i.e., NTproBNP) that have been demonstrated to increase the risk of incident HF [7, 8].

We defined MS at the V1 and V4 examination based on the International Diabetes Federation's definition. The International Diabetes Federation defines people with MS as individuals with an elevated waist circumference (≥94 cm for men; ≥80 cm for women) and two of the following: raised triglycerides (>150 mg/dL [1.7 mmol/L]), reduced HDL-C (<40 mg/dL [1.0 mmol/L] in men; <50 mg/dL [1.3 mmol/L] in females; or on therapy for reduced HDL-C), hypertension (systolic ≥130 mm Hg; diastolic ≥80 mm Hg; or on antihypertensive treatment), and elevated fasting glucose (≥100 mg/dL [5.6 mmol/L]; or on treatment for elevated glucose) [3].

For the primary analysis, we evaluated the association between MS and preclinical HF among individuals who were adults at the V1 examination. We utilized age- and sex-adjusted logistic regression and inverse probability weighting models. That is, among patients at V4, the inverse probability of treatment (i.e., exposure) was computed with a propensity score that was based on a logistic model adjusted for age and sex. The inverse probability of treatment was then balanced across the three groups (i.e., baseline MS, developed MS, and absent MS) to minimize between-group clinical differences and ensure exchangeability. The association between the three groups and preclinical HF was then evaluated utilizing a generalized logistic regression model. Sensitivity analyses removing the NTproBNP criterion from the preclinical HF definition were also conducted. A global *p* value comparing the three groups' associations with preclinical HF was computed in a multinomial weighted regression model. All analyses were conducted with SAS (v9.0).

Biological samples were obtained by venipuncture at the Nancy Hospital Center's Centre d'Investigation Clinique. Using sample-handling protocols, serum and plasma samples were obtained. All samples were then preserved at temperatures ranging from −80°C to −196°C in a central biobank. NTproBNP was then quantified utilizing an electrochemiluminescence immunoassay (Elecsys System, Roche Diagnostics, Basel, Switzerland).

## Results

**Fig. 1:**
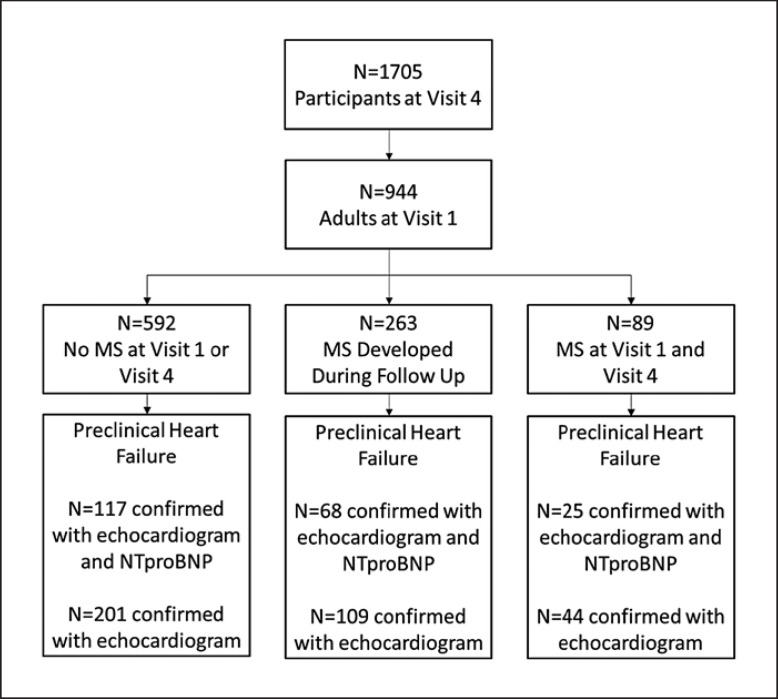
Consort diagram for the STANISLAS cohort.

The median follow-up time was 17 years (1993–2016). Among the 1,705 participants with V4 examinations between 2011 and 2016, only 944 individuals were adults at the V1 examination (Fig. 1). Among these 944 individuals, 872 (or 92% of the adult population) had echocardiograms conducted at V4, and of this subpopulation, 408 people had available data for NTproBNP at the same visit.

Overall, 89 (9%) patients had baseline MS and 263 (28%) patients developed MS during follow-up. At the V1 examination, 5% of study participants had a BMI >30 kg/m^2^. At the V4 examination, study participants with baseline MS were more likely to be older (63 vs. 61 vs. 59 years of age), male (73% vs. 55% vs. 45%), have elevated waist circumference (110 vs. 99 vs. 87 cm), hypertension (79% vs. 53% vs. 16%), and have elevated fasting glucose (28% vs. 13% vs. 1.4%; Table 1) compared to participants who developed MS and participants who had no MS at baseline, respectively.

**Fig. 2:**
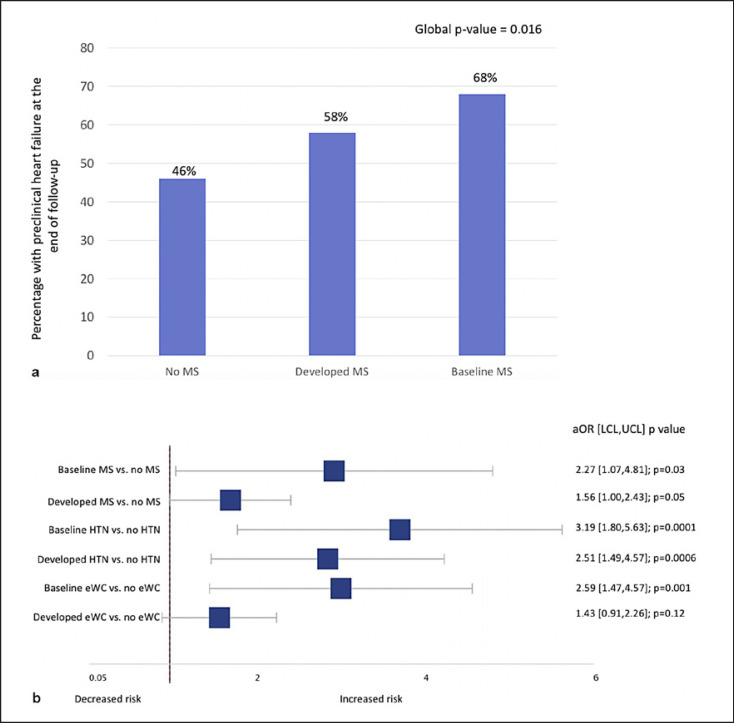
**a** Presence of preclinical HF at the end of follow-up. **b** aOR for the risk of preclinical HF at the end of follow-up. MS, metabolic syndrome; HTN, hypertension; eWC, elevated waist circumference; aOR, adjusted odds ratio; LCL, lower confidence limit; UCL, upper confidence limit.

In total, 210 of the 408 (51.4%) patients with complete echocardiographic and biomarker data developed preclinical HF (Fig 1). Compared to patients without baseline MS, the risk of preclinical HF was numerically increased both among patients who developed incident MS (adjusted odds ratio [aOR] 1.56, 95% CI: 1.00–2.43, *p* = 0.05) and patients who had MS at baseline (aOR 2.27, 95% CI: 1.07–4.81, *p* = 0.03; Fig. 2). Regarding the metabolic determinants of MS, the risk of preclinical HF was elevated in individuals with hypertension (aOR 3.19, 95% CI: 1.80–5.63) and elevated waist circumference (aOR 2.59, 95% CI: 1.47–4.57; Fig. 2). However, on evaluation, the other metabolic determinants of MS including elevated triglycerides, high-density lipoprotein, or fasting glucose were not associated with preclinical HF.

In the sensitivity analysis, 354 of the 872 (40.6%) patients developed preclinical HF with respect to the definition that omitted NTproBNP. Consistent results for the risk of preclinical HF were seen in this sensitivity analysis. Compared to patients without baseline MS, the risk of preclinical HF was numerically increased both among patients who developed incident MS (aOR 1.29, 95% CI: 0.94–1.78, *p* = 0.11) and among patients who had MS at baseline (aOR 2.27, 95% CI: 1.07–2.81, *p* = 0.04). Adjusting for BMI in addition to age and sex attenuated the significant association of baseline MS and preclinical HF (aOR 1.15; 95% CI: 0.49–2.71; *p* = 0.75), but a numerically increased risk was still observed.

## Discussion

After a median of 17 years of follow-up in our cohort of patients initially free of cardiovascular disease, baseline MS and incident MS were shown to be associated with an increased risk of preclinical HF. In addition, chronic exposure to an elevated waist circumference and hypertension (reflecting an overweight-hypertensive phenotype) appeared to be the metabolic determinants most associated with preclinical HF development.

Our analysis extends on prior work from cohorts with older patients with greater degrees of cardiovascular comorbidities. A prior analysis of 51,451 patients in the Women's Health Initiative, Multi-Ethnic Study of Atherosclerosis (MESA), and CHS suggested a dose-response relationship between BMI and the risk of HF with preserved ejection fraction (*p* trend HR <0.0001) [9]. In the MESA study, the risk of future cardiovascular disease (including clinically confirmed HF) was increased in patients with baseline obesity who developed MS compared to individuals without elevated BMI (aOR 1.60; 95% CI: 1.14–2.25) [10]. The combination of core laboratory-reported echocardiograms within a homogeneous patient cohort and long length of follow-up supports a robust assessment of the role of MS in the genesis of preclinical HF.

Our study had some limitations. First, our analysis did not assess the impact of diet and physical activity on modifying the relationship between MS and preclinical HF. As a result, the impact of diet and physical activity on HF development warrants further evaluation. Second, prior analyses have identified a poor association between biomarkers (e.g., NTproBNP) and structural heart disease; as a result, the use of natriuretic peptides in our definition of preclinical HF may have limited the analysis. Nevertheless, when we removed NTproBNP from the definition, the association between baseline MS and preclinical HF was still observed [11]. Third, the use of indexed left atrial volume or left ventricular mass, especially among patients with obesity, may have underestimated the presence of preclinical HF. Likewise, among patients who have atrial fibrillation or who are athletes, the prevalence of preclinical HF could have been overestimated. Fourth, the number of participants who had available data on NTproBNP and echocardiographic data (*n* = 408; 46.8%) was smaller than the population of adult patients with echocardiographic data alone (*n* = 872). Nevertheless, the association between baseline MS and preclinical HF was consistent in sensitivity analyses where NTproBNP was removed from the preclinical HF definition. Finally, there was no further follow-up available beyond V4 to enable analyses evaluating the association between MS and clinically confirmed acute HF.

Given the high risk of mortality when patients with MS or elevated fasting glucose become established with HF [12], HF prevention remains an important public health concern. Evaluation of early aggressive lifestyle, including diet and exercise, may help prevent preclinical and eventual HF development. Dietary strategies to reduce weight, especially among individuals who are obese and hypertensive, may represent a strategy for HF prevention [13]. Furthermore, the evaluation of various medical interventions such as blood pressure control or use of sodium-glucose cotransporter-2 inhibitors [14] among patients free of cardiovascular disease with an obese-hypertensive phenotype to prevent future HF development is warranted.

## Conclusion

Our study demonstrated that after 17 years of follow-up, patients with baseline MS, especially those with obese-hypertensive phenotypes, are at increased risk of developing preclinical HF. As a result, the primary prevention of preclinical HF should be an important public health focus given the high risk of mortality when patients with metabolic aberrations are diagnosed with clinically confirmed HF. Evaluating the preventive efficacy of early aggressive lifestyle modification and medical intervention among patients free of cardiovascular disease with obese-hypertensive phenotypes may be warranted.

## Statement of Ethics

All STANISLAS participants consented to having their data utilized for subsequent secondary analyses. As a result, no separate ethics approval was needed for the present study. The local Ethics Committee in Nancy, France (Comité de Protection des Personnes Est III-Nancy-France) approved the initial research protocol (ClinicalTrials.gov identifier NCT01391442).

## Conflict of Interest Statement

A.S. reports receiving support from the Fonds de Recherche Du Quebec Sante (FRQS) Junior 1 clinician scientist award, Takeda, Roche Diagnostics, BMS-Pfizer and is funded by the Alberta Innovates Health Solution Clinician Scientist Fellowship. A.R. reports receiving funding from McGill University's Graduate Excellence Award in Medicine & Ethelwyn and Ernie Nyman Fellowship. P.R. reports receiving consulting fees from Novartis, NovoNordisk, Relypsa, AstraZeneca, Grünenthal, Idorsia, Stealth Peptides, Fresenius, and Vifor; lecture fees from Bayer and CVRx; and he is the cofounder of CardioRenal. F.Z. reports consulting fees for serving on the board of Boston Scientific; consulting fees from Novartis, Takeda, AstraZeneca, Boehringer Ingelheim, GE Healthcare, Relypsa, Servier, Boston Scientific, Bayer, Johnson & Johnson, and Resmed; and speaking fees from Pfizer and AstraZeneca. There are no other conflicts to report.

## Funding Sources

The STANISLAS study is sponsored by Nancy CHRU. This work is supported by the French Ministry of Health “Programme Hospitalier de Recherche Clinique Inter regional 2013,” by the Contrat de Plan Etat-Lorraine and FEDER Lorraine, and a public grant overseen by the French National Research Agency (ANR) as part of the second “Investissements d'Avenir” program FIGHT-HF (reference: ANR-15-RHU-0004), and by the French PIA project “Lorraine Université d'Excellence,” reference ANR-15-IDEX-04-LUE. It is also supported by the European Fibro-Targets Project (Grant agreement No. SP7#602904), European HOMAGE project (Grant agreement No. Heart “Omics” in Ageing, 7th Framework Program Grant # 305,507), the MEDIA project (Européen “Cooperation”—Theme “Health”/FP7-HEALTH-2010-single-stage (reference: 261409), FOCUS-MR (reference: ANR-15-CE14-0032-01), ERA-CVD EXPERT (reference: ANR-16-ECVD-0002-02), and the Fondation de Recherche en Hypertension Artérielle.

## Author Contributions

The study was conceptualized by A.S., and F.Z. A.S. and J.P.F. contributed to the study methodology. A.S. and J.P.F. conducted the study investigation and contributed to the writing (original draft) of the manuscript. A.S., A.R., J.P.F., J.L.M., E.B., N.G., P.R., and F.Z. contributed to the writing (review and editing) of the manuscript. F.Z. supervised the study. We attest that all authors have reviewed and approved the article prior to its submission.

## Data Availability Statement

The data that support the findings of this study are not publicly available because of their containing information that could compromise the privacy of research participants but are available from the corresponding author (F.Z.) upon reasonable request.

## Figures and Tables

**Table 1:** Characteristics of the STANISLAS population based on metabolic syndrome status at the final visit

Characteristics	No metabolic syndrome at baseline or follow-up (*n* = 592)	Incident metabolic syndrome developed during follow-up (*n* = 263)	Metabolic syndrome at baseline and follow-up (*n* = 89)	*p* value
*Clinical characteristics*				
Age, years	59 (56–62)	61 (58–64)	63 (59–65)	<0.0001
Women, *n* (%)	328 (55.4)	118 (44.9)	24 (27.0)	<0.0001
Height, m	1.67±0.09	1.67±0.09	1.70±0.09	0.020
Weight, kg	68 (60–78)	81 (72–89)	93 (80–104)	<0.0001
BMI, kg/m^2^	24.4 (22.4–26.8)	28.4 (26.3–31.1)	31.9 (28.8–34.9)	<0.0001
Waist circumference, cm	87±11	99±10	110±12	<0.0001
Current smoker, *n* (%)	73 (12.3)	30 (11.4)	10 (11.2)	0.93
eGFR, mL/min/1.73 m^2^	92 (82–98)	90 (79–97)	89 (77–98)	0.18
eGFR <60 mL/min/1.73 m^2^, *n* (%)	9 (1.5)	6 (2.3)	3 (3.4)	0.42
Total cholesterol, g/L	2.27±0.35	2.17±0.40	1.96±0.45	<0.0001
HDL, g/L	0.63±0.14	0.53±0.12	0.47±0.10	<0.0001
LDL, g/L	1.45±0.31	1.35±0.34	1.19±0.37	<0.0001
Hypercholesterolemia treatment, *n* (%)	58 (9.8)	125 (47.5)	51 (57.3)	<0.0001
Fasting glycemia, g/L	0.88 (0.83–0.94)	0.98 (0.89–1.05)	1.04 (0.95–1.20)	<0.0001
Diabetes, *n* (%)	8 (1.4)	34 (12.9)	25 (28.4)	<0.0001
Diabetes treatment, *n* (%)	5 (0.8)	24 (9.1)	20 (22.5)	<0.0001
Antihypertensive treatment, *n* (%)	93 (15.7)	139 (52.9)	70 (78.7)	<0.0001
Office SBP, mm Hg	125±14	135±16	138±17	<0.0001
Office DBP, mm Hg	73±8	77±9	77±9	<0.0001
Nocturnal SBP, mm Hg	110±10	115±11	118±12	<0.0001
Diurnal SBP, mm Hg	124±10	128±11	132±12	<0.0001
24-h SBP, mm Hg	119±10	123±10	127±12	<0.0001
NTproBNP, pg/mL*	54 (34–83)	51 (30–120)	38 (27–121)	0.59
*Echocardiographic parameters*				
LVEF, %	65±6	66±6	65±8	0.23
LV mass/BSA, g/m^2^	74 (64–88)	82 (69–93)	88 (73–100)	<0.0001
LAV/BSA, mL/m^2^	22 (17–27)	23 (18–28)	23 (18–28)	0.053
E wave, cm/s	65±14	66±16	67±14	0.41
A wave, cm/s	64±15	72±17	76±15	<0.0001
Septal Ea velocity, cm/s	9.0±2.3	8.1±2.3	7.6±1.9	<0.0001
Lateral Ea velocity, cm/s	11.4±3.2	10.5±2.7	9.6±2.7	<0.0001
Deceleration time, ms	218±52	217±60	230±58	0.12
A wave duration, ms	137±27	143±30	144±23	0.004
Pulmonary reversal A wave duration, ms	109±20	111±19	114±17	0.072

Continuous variables are described as mean ± SD or as median (IQR) according to their normality, and categorical variables are described as frequencies (percentage). Preclinical HF (1) was defined only on patients with an NTproBNP measurement. Subjects had a preclinical HF if they had DD grade II or III and/or LVH and/or LAE and/or high NTproBNP. Preclinical HF (2) was defined in all the population and excluding the NTproBNP criterion. Subjects had preclinical HF if they had DD grade II or III and/or LVH and/or LAE. BMI, body mass index; eGFR, estimated glomerular filtration rate; HDL, high-density lipoprotein; LDL, low-density lipoprotein; SBP, systolic blood pressure; DBP, diastolic blood pressure; LVEF, left ventricular ejection fraction; LV, left ventricular; LAV, left atrial volume; LVH, left ventricular hypertrophy; LAE, left atrial enlargement.

* *n* = 447 with NTproBNP measurements.
